# A high throughput gas exchange screen for determining rates of photorespiration or regulation of C_4_ activity

**DOI:** 10.1093/jxb/eru238

**Published:** 2014-06-13

**Authors:** Chandra Bellasio, Steven J Burgess, Howard Griffiths, Julian M Hibberd

**Affiliations:** ^1^Department of Animal and Plant Sciences, University of Sheffield, Western Bank, Sheffield S10 2TN, UK; ^2^Department of Plant Sciences, University of Cambridge, Downing Street, Cambridge CB2 3EA, UK

**Keywords:** C4, C3, photosynthesis, RuBisCO, oxygenation, carboxylation, carbon concentration mechanism (CCM), *Cleome gynandra*, rice, maize, wheat, *Miscanthus*.

## Abstract

Large-scale research programmes seeking to characterize the C_4_ pathway have a requirement for a simple, high throughput screen that quantifies photorespiratory activity in C_3_ and C_4_ model systems. At present, approaches rely on model-fitting to assimilatory responses (*A*/*C*_i_ curves, PSII quantum yield) or real-time carbon isotope discrimination, which are complicated and time-consuming. Here we present a method, and the associated theory, to determine the effectiveness of the C_4_ carboxylation, carbon concentration mechanism (CCM) by assessing the responsiveness of *V*_O_/*V*_C_, the ratio of RuBisCO oxygenase to carboxylase activity, upon transfer to low O_2_. This determination compares concurrent gas exchange and pulse-modulated chlorophyll fluorescence under ambient and low O_2_, using widely available equipment. Run time for the procedure can take as little as 6 minutes if plants are pre-adapted. The responsiveness of *V*_O_/*V*_C_ is derived for typical C_3_ (tobacco, rice, wheat) and C_4_ (maize, *Miscanthus*, cleome) plants, and compared with full C_3_ and C_4_ model systems. We also undertake sensitivity analyses to determine the impact of *R*_LIGHT_ (respiration in the light) and the effectiveness of the light saturating pulse used by fluorescence systems. The results show that the method can readily resolve variations in photorespiratory activity between C_3_ and C_4_ plants and could be used to rapidly screen large numbers of mutants or transformants in high throughput studies.

## Summary

A rapid approach to determine photorespiratory activity in plants suitable for analysis of photosynthetic mutants, C_3_–C_4_ intermediates, as well as lines with a weak glycine shuttle.

## Introduction

In most photosynthetic organisms Ribulose Bisphosphate Carboxylase Oxygenase (RuBisCO) catalyses the first key step in carbon assimilation, reacting ribulose-1,5-bisphosphate with CO_2_ to produce two molecules of 3-phosphoglycerate (PGA). Oxygen competitively inhibits this reaction and leads to the synthesis of the 2-carbon compound phosphoglycollate, which is recycled to PGA (consuming ATP, and then NADPH) and CO_2_ by the photorespiratory cycle (Yoshimura *et al.*, 2004; Sage *et al.*, 2012). The result of photorespiration is a noticeable carbon loss and a consequent metabolic cost for carbon recapture and for the recycling of photorespiratory intermediates (Ehleringer and Pearcy, 1983; Pearcy and Ehleringer, 1984; Eckardt, 2005). Many plants have evolved strategies to reduce photorespiration by increasing the level of CO_2_ around RuBisCO, including both crassulacean acid metabolism (CAM) and the C_4_ photosynthetic pathway (Dodd *et al.*, 2002; Sage, 2004; Sage *et al.*, 2011; Osborne and Sack, 2012; Griffiths *et al.*, 2013; Owen and Griffiths, 2013). C_4_ photosynthesis is most often based on a two-celled carbon concentrating mechanism, where HCO_3_^–^ is first fixed into the four-carbon compound oxaloacetic acid (OAA) in the mesophyll by phospho*enol*pyruvate carboxylase (PEPC). OAA is then reduced to malate or transaminated to aspartate and the resulting C_4_-(amino)acid is shuttled into the bundle sheath (BS), where it is decarboxylated, releasing CO_2_ for refixation by RuBisCO (Hibberd and Covshoff, 2010; Bellasio and Griffiths, 2014c).

Although the enzymes catalysing the core C_4_ carbon concentration mechanism (CCM) are well characterized (Kanai and Edwards, 1999), many of the genes responsible for the accompanying anatomical alterations or for generating and maintaining expression of the C_4_ cycle genes (Hibberd *et al.*, 2008; Langdale, 2011) have yet to be identified. One approach that is increasingly proving useful to identify candidate genes underlying the C_4_ pathway is comparative transcriptomics of samples either undergoing C_3_ or C_4_ photosynthesis (Bräutigam *et al.*, 2011; Gowik *et al.*, 2011; John *et al.*, 2014), or tissues in the process of inducing the full C_4_ system (Li *et al.*, 2010; Pick *et al.*, 2011; Chang *et al.*, 2012; Wang *et al.*, 2013). Because stable transformation of C_4_ species is typically time-consuming, introduction of RNA interference constructs via a transient *Agrobacterium tumefaciens*-based system would be very helpful in screening these candidates being generated from transcriptomics.

At present, techniques used to screen for mutants possessing defective, or enhanced CCM characteristics are time-consuming (Table 1). Analysing the response of assimilation (*A*) to decreasing CO_2_ concentration in the substomatal cavity (*C*_i_), as *A*/*C*_i_ curves (Long and Bernacchi, 2003; Yin *et al.*, 2011a) can take 45 minutes per replicate leaf, and an appropriate model, which may require *a priori* knowledge of species-specific limitations (Laisk and Edwards, 2000; von Caemmerer, 2000, 2013; Yin and Struik, 2009; Yin *et al.*, 2009; Yin *et al.*, 2011b). ^13^C/^12^C discrimination during photosynthesis (Evans *et al.*, 1986) can also be used, and a comparison with stomatal conductance allows the internal mesophyll conductance, or extent of CCM or PEPC activity, to be resolved (Meyer *et al.*, 2008; Kromdijk *et al.*, 2010; Pengelly *et al.*, 2010; Bellasio and Griffiths, 2014a, b, c). However, this latter technique is sensitive, and requires either off-line sample preparation for mass spectrometric analyses or specialized laser equipment which is not readily available (Table 1).

**Table 1.** Comparison between methods screening for activity of a functional CCM

Method	Advantages and limitations	Reference
Dry matter isotopic discrimination	*Specialized equipment *Integrates the isotopic signal throughout growth *Cannot resolve transient changes in assimilatory physiology	Cernusak *et al*. (2013)
On line isotopic discrimination	*Laser is no longer commercially available *Maintenance costs of isotope ratio mass spectrometer *Need of highly skilled operator *Difficult computation and parameterization	Evans *et al.* (1986); Bellasio and Griffiths (2014b); von Caemmerer *et al*. (2014)
*A*/*C*_i_ curves	*Requires *a priori* knowledge of the limitations underpinning each part for the *A*/*C*_i_ curve for correct model fitting *Result may depend on experimental routine	Long and Bernacchi (2003); Yin *et al.* (2009)
Gas exchange and fluorescence	*Requires initial response curve for parameterisation *Requires model fitting	Long and Bernacchi (2003); Martins *et al.* (2013)
O_2_ sensitivity of carboxylation efficiency	*Delicate experimental routine	Laisk *et al.* (2002); Yin *et al.* (2009)
Assimilation increase under low O_2_	*Ease of determination *Ignores the effect of changing O_2_ concentration on *Y(II)*	Sharkey (1988); Ripley *et al.* (2007)
Gas exchange and fluorescence	*Rapid (6 minutes) *Widely available equipment *Independent of leaf size *Ease of determination and calculation *Does not require fitting or parameterisation *Assessment under growth conditions	This study

In this paper we describe a novel method, and present the associated theory, to determine rates of photorespiration from instantaneous rates of RuBisCO carboxylation and oxygenation. The approach compares concurrent gas exchange and pulse-modulated chlorophyll fluorescence measurements under ambient and low O_2_. Under these non-photorespiratory conditions assimilation (*A*) increases, because RuBisCO competitive inhibition from O_2_ is reduced. In contrast, *Y(II)* decreases because the demand for NADPH associated with photorespiratory by-product cycling (and reduction) is lower, and cannot entirely be offset by the increase in *A*. The new method combines developments in approaches using gas exchange (Sharkey, 1988; Long and Bernacchi, 2003; Ripley *et al.*, 2007) and the quantitative interpretation of quantum yield (Yin *et al.*, 2004, 2009, 2011b; Yin and Struik, 2009, 2012; Bellasio and Griffiths, 2014b). This new method can be performed with off-the-shelf commercial equipment, which is generally available in ecophysiology laboratories. The procedure takes as little as 6 minutes to perform if plants are pre-adapted, making it significantly faster than *A*/*C*_i_ curves and potentially useful as a high-throughput approach for assessing C_4_ activity in mutant screens, the progeny from C_3_–C_4_ crosses or C_3_–C_4_ intermediates.

## Materials and methods

### Plants

Plants of *Miscanthus* (*Miscanthus giganteus*), cleome (*Cleome gynandra*), maize (*Zea mays* L.), wheat (*Triticum aestivum* L.), tobacco (*Nicotiana tabacum* L.), and rice (*Oryza sativa* L.) were grown at the Plant Growth Facility located at the University of Cambridge Botanic Garden in controlled environment growth rooms (Conviron Ltd, Winnipeg, Canada) set at 16h day length, temperature of 25 °C/23 °C (day/night), 40% relative humidity, and photosynthetic photon flux density (PPFD)=300 μmol m^–2^ s^–1^. Plants were manually watered daily, with particular care to avoid overwatering.

### Gas exchange measurements with concurrent PSII yield

**Fig. 1.**
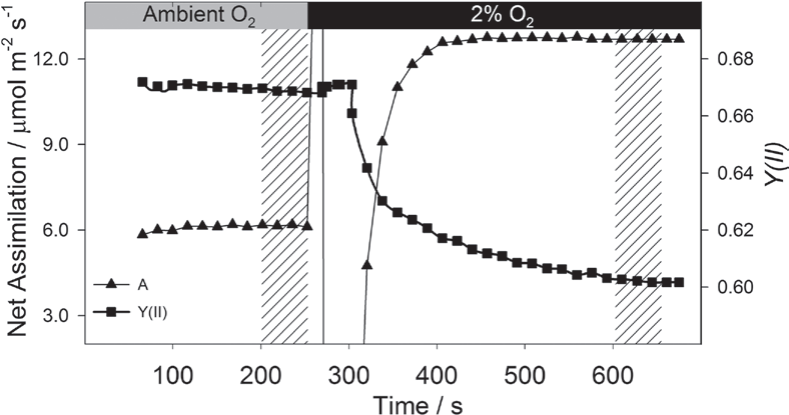
Summary of experimental approach. One representative dataset from C_3_ tobacco is presented. Once stable assimilatory conditions are reached, a first set of data are recorded (left hatched area). The background gas is then switched from ambient to 2% O_2_. After a suitable acclimation time to allow flushing of the cuvette and reacclimation (c. 6min), a second set of data are recorded (right hatched area). The response of assimilation (triangles) and Photosystem II yield *Y(II)* (squares) during the experiment are shown.

Measurements were performed with an infra-red gas analyser (IRGA, a LI6400XT, LI-cor, USA), fitted with a 6400–40 leaf chamber fluorometer. The IRGA was fed with CO_2_ (through the IRGA gas mixing unit) and ambient air. Gas flow was set at 150 μmol s^–1^. Reference CO_2_ was set at 200 μmol mol^–1^ (Figure 1 and Table 1) or set alternatively at 400, 300, 200, 150, 100, and 50 μmol s^–1^ (Figure 3). Block temperature was controlled at 35 °C. The fluorometer was set to multiphase pulse with factory setting, target intensity=10 and ramp depth=40% (Loriaux *et al.*, 2013). A portion of a light-adapted leaf was clamped in the cuvette. The leaf was allowed to reach stable photosynthetic conditions under PPFD=300 μmol m^–2^ s^–1^ (factory setting: 90% red, 10% blue). Photosynthesis was measured every 10 s for 30 s (the three values were then averaged) and a multiphase pulse was applied for the determination of *Y(II)*. A humidified 2% O_2_/N_2_ gas (pre-mixed, BOC, Guilford, UK) was switched to supply the inlet of the IRGA. The gas was allowed to completely flush the cuvette (c. 6min). Photosynthesis was measured every 10 s for 30 s (the three values were then averaged) and a multiphase pulse was applied for the determination of *Y(II)*. Light was turned off, the inlet was fed with ambient air, the reference CO_2_ was set at 500 μmol mol^–1^, similar to the lab CO_2_ concentration (c. 550 μmol mol^–1^) to minimize the errors caused by CO_2_ leakage (Boesgaard *et al.*, 2013), and flow was set to 40 μmol s^–1^. Once the cuvette had been flushed, and the signal stabilised (c. 5min), respiration was measured every 10 s for 2min (the values were then averaged). *C*_a_ was not adjusted to account for changes in stomatal conductance or for the control of *C*_i_ during this procedure. This avoided the need for IRGA recalibration as the *Y(II)* measurements are independent of *C*_i_. The measured *A* and *Y(II)* under low and ambient O_2_, together with an estimate of *R*_LIGHT_ (see below), were used to determine RuBisCO rate of carboxylation (*V*_C_), RuBisCO rate of oxygenation (*V*_O_), and the rate of photorespiratory CO_2_ evolution in the light (*F*).

### Theory

RuBisCO catalyses two reactions: a carboxylase reaction whereby Ribulose BisPhosphate (RuBP) is carboxylated to form two molecules of phosphoglyceric acid (PGA), and an oxygenase reaction whereby RuBP is oxygenated to form one PGA and one glycollate molecule. Each carboxylase event requires 2 NADPH for the reduction of the 2 PGA molecules formed. Each oxygenase event requires 1 NADPH for the reduction of the PGA directly produced by RuBisCO, 0.5 NADPH to recycle glycollate, and 0.5 NADPH to reduce the PGA regenerated, which total 2 NADPH (Bellasio and Griffiths, 2014c). The overall NADPH demand, at steady-state, equals the total photosynthetic NADPH production rate *J*_NADPH_ (Yin *et al.*, 2004; Yin and Struik, 2012):

$$J_{\text{NADPH}}=2V_{\text{C}}+2V_{\text{O}}$$ (1)

Where *J*_NADPH_ is the total NADPH produced for photosynthesis, *V*_C_ is RuBisCO carboxylation rate, and *V*_O_ is RuBisCO oxygenation rate. Notably, this reducing power requirement is the same for all types of photosynthesis, as active types of CCM require additional ATP but not NADPH. In line with von Caemmerer (2000) equation 1 assumes that PGA is entirely reduced, and therefore the small quantity of PGA consumed by respiration ($\frac{1}{3}$ *R*_LIGHT_) is neglected, in fact under growth light irradiance 2*V*_C_+2*V*_O_>>$\frac{1}{3}$ *R*_LIGHT_, unless at very low irradiances, see equation 7 in Bellasio and Griffiths (2014c).

Although the carboxylation reaction of RuBisCO consumes CO_2_, the regeneration of glycollate releases 0.5 CO_2_ for each oxygenase catalytic event. CO_2_ is also produced by light respiration, a process which is active during photosynthesis to support basal metabolism. The net assimilation rate (*A*, which is the quantity measured through gas exchange) results from summing the CO_2_ consumed by RuBisCO, the CO_2_ produced by glycollate regeneration and the CO_2_ produced by respiration:

$$A=V_{\text{C}}-\frac{1}{2}V_{\text{O}}-R_{\text{LIGHT}}$$ (2)

Where *A* is net CO_2_ assimilation, *R*_LIGHT_ is respiration in the light and other variables were previously defined. Notably, this equation is universal for all types of photosynthesis (von Caemmerer, 2013).

For the definition of gross assimilation (*GA* = *A* + *R*_LIGHT_), equation 2 can be rearranged:

$$V_{\text{C}}=GA+\frac{1}{2}V_{\text{O}}$$ (3)

Equation 1 and 3 can be combined to give:

$$V_{\text{O}}=\frac{J_{\text{NADPH}}-2GA}{3}$$ (4)

The rate of photorespiratory CO_2_ evolution, *F* can be calculated as: (von Caemmerer, 2013)

$$F=\frac{1}{2}V_{\text{O}}$$ (5)

Under low O_2_, *V*_O_ can be approximated to ≈0, hence, from equation 4:

$$J_{\text{NADPH Low O}2}=2GA_{\text{Low O}2}$$ (6)

Which is valid when *V*_O_≈0.

NADPH is produced through linear electron flow. Independently from where this reaction is located (e.g. in mesophyll cells), electrons are invariably extracted from water by PSII (Yin and Struik, 2012), therefore *J*_NADPH_ is proportional to *Y(II)* (Yin and Struik, 2012). This allows *J*_NADPH_ to be calculated under photorespiratory conditions using the information derived under non-photorespiratory conditions, and can be expressed as (Bellasio and Griffiths, 2014b):

$$J_{\text{NADPH}}=J_{\text{NADPH Low O}2}\frac{Y(II)}{Y{\left(II\right)}_{\text{Low O}2}}$$ (7)

Where *J*_NADPH_ and *Y(II)* refer to ambient O_2_ conditions. Equation 7 has been validated in C_3_ and C_4_ plants (Yin *et al.*, 2009, 2011b; Bellasio and Griffiths, 2014b, c) but it is worth noting that equation 7 is a mathematical simplification and holds true when: (i) photorespiration is negligible under non-photorespiratory conditions, which is a widely used simplification; (ii) *R*_LIGHT_ does not vary between low and ambient O_2_—this is also a fair assumption because any O_2_ effect is generally negligible (Badger, 1985; Gupta *et al.*, 2009); (iii) the allocation to alternative sinks (non-assimilatory and non-photorespiratory) is proportional to *Y(II)*. This is the normal case in C_4_ plants where the relationship between *Y(II)* and *Y(CO*_2_) has a null intercept (Edwards and Baker, 1993). When that is not the case, for instance when the allocation to alternative sinks is constant, equation 7 would also hold true if the allocation to alternative sinks is small compared with *Y(II)*. This is the normal case in C_3_ plants (Valentini *et al.*, 1995; Martins *et al.*, 2013). Should the allocation to alternative sinks be large, equation 7 would still hold true mathematically when $\frac{Y(II)}{Y{\left(II\right)}_{\text{Low O}2}}$ is close to the unity. The implications for method accuracy are detailed in the discussion.

Equation 3, 4, 6, and 7 can be combined to obtain:

$$\frac{V_{\text{O}}}{V_{\text{C}}}=\frac{2GA_{\text{Low O}2}\frac{Y(II)}{Y{\left(II\right)}_{\text{Low O}2}}-2GA}{GA_{\text{Low O}2}\frac{Y(II)}{Y{\left(II\right)}_{\text{Low O}2}}+2GA}$$ (8)

Which expresses the RuBisCO rate of oxygenation relative to carboxylation. The influence on the quality of *R*_LIGHT_ estimate on *V*_O_/*V*_C_ is described in the discussion, together with the other factors influencing the results.

### Modelling C_3_ and C_4_ *V*_O_/*V*_C_

The data obtained for tobacco and maize were compared with a simulated *V*_O_/*V*_C_ based on the validated von Caemmerer models for C_3_ and C_4_ photosynthesis. Briefly, for tobacco, the response of *A* to *C*_i_ was modelled using the quadratic equation (Table 3, equation 9) proposed by Ethier and Livingston (2004), which takes into account mesophyll conductance to CO_2_. The CO_2_ concentration at the site of carboxylation *C*_C_ was then calculated through the supply function of mesophyll (equation 10), and, finally *V*_O_/*V*_C_ was simulated from the kinetic properties of RuBisCO and the ratio between *C*_C_ and the O_2_ concentration at the site of carboxylation (equation 11). For maize (Table 4), firstly we simulated the responses of *V*_P_ and *A* to decreasing *C*_i_, using the equations for the enzyme-limited model for C_4_ photosynthesis (equation 12 and 16, respectively). These were used to simulate the CO_2_ and O_2_ concentration in the bundle sheath (equation 13 and 14, respectively), the ratio of which, together with RuBisCO specificity, was used to simulate *V*_O_/*V*_C_ (equation 15 and 17).

**Table 2.** Example of variability within populations and between populations displayed by plants with different pathways of assimilation *V*_O_/*V*_C_ was measured on species (*Miscanthus*, *Cleome gynandra*, maize, wheat, tobacco, and rice) under photosynthetic photon flux density (PPFD) of 300 μmol m^–2^ s^–1^, and *C*_a_=200 μmol mol^–1^.

Population	*n*	Mean *V*_O_/*V*_C_	Standard deviation	Coefficient of variation
*Miscanthus*	7	0.0504	0.0091	18%
*Cleome gynandra*	5	0.0852	0.0046	5.4%
Maize	4	0.0435	0.0074	17%
Wheat	3	0.522	0.071	14%
Tobacco	4	0.533	0.030	5.5%
Rice	4	0.569	0.037	6.5%

**Table 3.** Model for C_3_ photosynthesis

Symbol	Definition/calculation	Equation	Values/Units/References
*A*	Net Assimilation $A=\frac{-\;b\;+\;\sqrt{b^{2}-4ac}}{2a}$ where: $a\;=-\frac{1}{{\text{g}}_{\text{m}}};$ $b\;=\frac{\left(V_{\text{Cmax}}-R_{\text{LIGHT}}\right)}{g_{\text{m}}}+C_{\text{i}}+K_{\text{C}}(1+\frac{O}{K_{\text{O}}});$ $\begin{array}{l} c\;=R_{\text{LIGHT}}\left(C_{\text{i}}+K_{\text{C}}(1+\frac{O}{K_{\text{O}}})\right)\\ \;-V_{\text{Cmax}}\left(C_{\text{i}}-\Gamma^{*}\right) \end{array}$	(9)	Ethier and Livingston (2004)
*C*_c_	CO_2_ partial pressure at the site of carboxylation $\;C_{\text{c}}\;=C_{\text{i}}-\frac{A}{{\text{g}}_{\text{m}}}$	(10)	μbar
*C*_i_	CO_2_ concentration in the intercellular spaces as calculated by the IRGA.		μmol mol^–1^ (Li-cor 6400 manual equation 1–18)
*g*_m_	Mesophyll conductance to CO_2_		0.25mol m^–2^ s^–1^ bar^–1^ (Ethier and Livingston, 2004)
*K*_C_	RuBisCO Michaelis-Menten constant for CO_2_		319.3 μbar (Ethier and Livingston, 2004)
*K*_O_	RuBisCO Michaelis-Menten constant for O_2_		277100 μbar (Ethier and Livingston, 2004)
*O*	O_2_ partial pressure at the site of carboxylation		200000 μbar
*R*_LIGHT_	Respiration in the light		0.63 μmol m^–2^ s^–1^
*V*_Cmax_	Maximum RuBisCO carboxylation rate		34.7 μmol m^–2^ s^–1^ (Ethier and Livingston, 2004)
*V*_O_ */V*_C_	$\frac{V_{\text{O}}}{V_{\text{C}}}=\frac{V_{\text{Omax}}{\text{K}}_{\text{C}}}{V_{\text{Cmax}}{\text{K}}_{\text{O}}}\;\frac{O}{C_{\text{C}}}$	(11)	equation 2.16 in (von Caemmerer, 2000)
*V*_Omax_	Maximum RuBisCO oxygenation rate		13.25 μmol m^–2^ s^–1^ (Ethier and Livingston, 2004)
Γ*	CO_2_ compensation point in absence of dark respiration		44 μbar

**Table 4.** Model for C_4_ photosynthesis

Symbol	Definition/calculation	Equation	Values/Units/References
*A*	Net Assimilation $A=\frac{-\;b\;-\;\sqrt{b^{2}-4ac}}{2a}$ where: $a\;=1-\frac{\alpha K_{\text{C}}}{0.047K_{\text{O}}}$ ; $b\;=-\left\{\left(V_{\text{P}}-R_{\text{M}}+g_{\text{BS}}{\text{C}}_{\text{M}}\right)+\left(V_{\text{Cmax}}-R_{\text{LIGHT}}\right)+g_{\text{BS}}{\text{K}}_{\text{C}}\left(1+\frac{O_{\text{M}}}{K_{\text{O}}}\right)+\frac{\alpha }{0.047}(\gamma^{*}\;V_{\text{Cmax}}+R_{\text{LIGHT}}\frac{K_{\text{C}}}{K_{\text{O}}})\right\};$ $c\;=\left(V_{\text{Cmax}}-R_{\text{LIGHT}}\right)\left(V_{\text{P}}-R_{\text{M}}+g_{\text{BS}}{\text{C}}_{\text{M}}\right)-(V_{\text{Cmax}}g_{\text{BS}}\gamma^{*}O_{\text{M}}+R_{\text{LIGHT}}g_{\text{BS}}{\text{K}}_{\text{C}}\left(1+\frac{O_{\text{M}}}{K_{\text{O}}}\right))$	(12)	Equation 4.21 in (von Caemmerer, 2000)
*C*_BS_	CO_2_ concentration in the bundle sheath $C_{\text{BS}\;}=\frac{\gamma^{*}O_{\text{BS}}+{\text{K}}_{\text{C}}\left(1+\frac{O_{\text{BS}}}{K_{\text{O}}}\right)\;\frac{A+R_{\text{LIGHT}}}{V_{\text{Cmax}}}}{1-\;\frac{A+R_{\text{LIGHT}}}{V_{\text{Cmax}}}}$	(13)	Equation 4.11 in (von Caemmerer, 2000)
*C*_M_	CO_2_ partial pressure in M (at the site of PEP carboxylation) $\;C_{\text{M}}\;={\text{C}}_{\text{i}}$		μbar
*C*_i_	CO_2_ concentration in the intercellular spaces as calculated by the IRGA		μbar
*g*_*BS*_	Bundle sheath conductance to CO_2_		0.005mol m^2^ s^–1^
*K*_C_	RuBisCO Michaelis-Menten constant for CO_2_		650 μbar (von Caemmerer, 2000)
*K*_O_	RuBisCO Michaelis-Menten constant for O_2_		450000 μbar (von Caemmerer, 2000)
*K*_P_	PEPC Michaelis-Menten constant		80 μbar (von Caemmerer, 2000)
*O*_BS_	O_2_ mol fraction in the bundle sheath cells (in air at equilibrium) $O_{BS}=O_{\text{M}}+\frac{\alpha A}{0.047g_{\text{BS}}}$	(14)	μmol mol^–1^ Equation 4.16 in (von Caemmerer, 2000)
*O*_M_	O_2_ partial pressure in the mesophyll cells (in air at equilibrium)		210000 μbar
*R*_LIGHT_	Respiration in the light, assumed to equal dark respiration		
*R*_M_	Mesophyll non photorespiratory CO_2_ production in the light *R*_M_ = 0.5 *R*_LIGHT_		μmol m^–2^ s^–1^ (von Caemmerer, 2000; Kromdijk *et al.*, 2010; Ubierna *et al.*, 2013)
*V*_Cmax_	Maximum RuBisCO carboxylation rate		60 μmol m^–2^ s^–1^ (von Caemmerer, 2000
*V*_O_ */V*_C_	$\frac{V_{\text{O}}}{V_{C}}=\frac{2\;\Gamma^{*}}{C_{\text{BS}}}$	(15)	Equation 4.8 in (von Caemmerer, 2000)
*V*_P_	PEP Carboxylation rate $V_{\text{P}}=\frac{C_{\text{M}}V_{\text{Pmax}}}{C_{\text{M}}+K_{\text{P}}}$	(16)	Equation 4.17 in (von Caemmerer, 2000)
*V*_Pmax_	Maximum PEPC carboxylation rate		120 μmol m^–2^ s^–1^ (von Caemmerer, 2000)
*α*	Fraction of PSII active in BS cells		0.15 (Edwards and Baker, 1993; von Caemmerer, 2000; Kromdijk *et al.*, 2010)
γ*	Half of the reciprocal of the RuBisCO specificity		0.000193 (von Caemmerer, 2000)
Γ*	CO_2_ compensation point in absence of dark respiration $\;\Gamma^{*}\;=\gamma^{*}O_{\text{BS}}$	(17)	Equation 4.9 in (von Caemmerer, 2000)

## Results

**Fig. 2.**
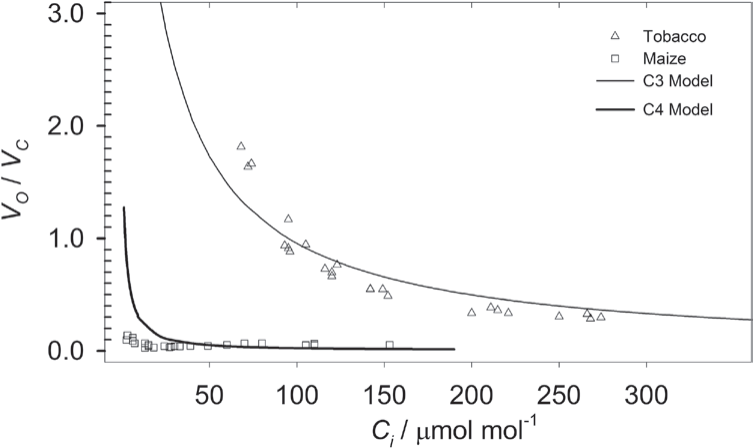
V_O_/*V*_C_ measured under different CO_2_ concentrations in the substomatal cavity (C_i_), obtained by imposing reference CO_2_ concentrations of 400, 300, 200, 150, 100, and 50 μmol mol^–1^ for C_3_ tobacco (triangles) and C_4_ maize (squares). Data are compared with simulated *V*_O_/*V*_C_ using the validated von Caemmerer C_3_ and C_4_ models (lines, see also Table 3 and 4). With decreasing *C*_i_, *V*_O_/*V*_C_ gets progressively higher in tobacco but it is only marginally affected in maize, CO_2_ concentration can therefore be used to control the resolution of the method. All data shown, *n*=4.

Figure 1 displays a typical primary data profile for a C_3_ tobacco leaf, showing the interaction between steady state assimilation (*A*) and quantum yield of PSII, *Y(II)*, during the transition from ambient to low O_2_ (21 to 2% O_2_), with hatched areas indicating the steady state conditions under which readings were taken to derive *V*_O_/*V*_C_. Under non-photorespiratory conditions, *A* increases because of the lower competitive inhibition of O_2_, whereas *Y(II)* decreases owing to the lower NADPH demand for photorespiratory by-product recycling and reduction. The experimental conditions were deliberately chosen to minimize reductions of quantum yield at saturating light (relatively low PPFD of 300 μmol m^–2^ s^–1^), and enhance photorespiratory responses to low O_2_ partial pressure (measurements at 200 μmol mol^–1^ CO_2_) (Fig. 1 and Table 2). Subsequently, *V*_O_/*V*_C_ was measured on C_3_ tobacco and C_4_ maize using different CO_2_ concentrations in the reference gas: 400, 300, 200, 150, 100, and 50 μmol mol^–1^ (Fig. 2) and results were compared with simulated values of *V*_O_/*V*_C_ generated with the validated von Caemmerer C_3_ and C_4_ models. To facilitate the comparison, data were plotted against the substomatal CO_2_ concentration *C*_i_. As expected, under decreasing *C*_i_, *V*_O_/*V*_C_ becomes progressively higher in tobacco but it is only marginally affected in maize. The measured data track the trend and magnitude of the theoretical curves in C_3_, whereas we could not capture the theoretical increase in *V*_O_/*V*_C_ expected when *C*_i_ was close to zero. This may be due to errors in the determination of *C*_i_ at very low stomatal conductance or to the simplifications used to resolve equation 7. Our data slightly underestimate *V*_O_/*V*_C_ derived using pulsed of ^13^C enriched CO_2_ (Busch *et al.*, 2013), which, however, lay above the curve simulated with the von Caemmerer C_3_ model (see Fig. 2).

Additional measurements were undertaken with the IRGA, including a recalibration procedure to account for the changing sensitivity to water vapour pressure after the transition to low O_2_, but stomatal conductance was reduced on average by 1% and internal CO_2_ concentration, *C*_i_, by 3 μmol mol^–1^ (data not shown). In the subsequent sections, primary data for *V*_O_/*V*_C_ determinations using this new method (calculated from equation 4) are initially presented for three representatives of C_3_ and C_4_ species. We then undertake a systematic error analysis of the method, to include the impact of biological and environmental variables. These include physiological components (*R*_LIGHT_) and *Fm′*, as well as light intensity and CO_2_ concentration used during experimentation.

### Variability between and within populations

Table 2 demonstrates that the method clearly discriminates between C_4_ species, possessing a functional CCM, and C_3_ species with higher rates of photorespiration. *V*_O_/*V*_C_ ranged from 0.0435 to 0.0852 for the representative C_4_ species, with coefficients of variation ranging from c. 15% down to 5% in *C. gynandra* (Table 2). For the C_3_ species, *V*_O_/*V*_C_ ranged from 0.522 to 0.569, with a low coefficient of variation in tobacco and rice around 6% (Table 2). The magnitude of the offset between C_3_ and C_4_ systems, if being used as a rapid screen, would allow changes in expression of C_4_ characteristics to be clearly resolved. Such an approach would then allow more detailed characterisation of selected transformants, C_2_, or C_3_–C_4_ intermediates to be undertaken.

### Accuracy of *R*_LIGHT_ estimates

**Fig. 3.**
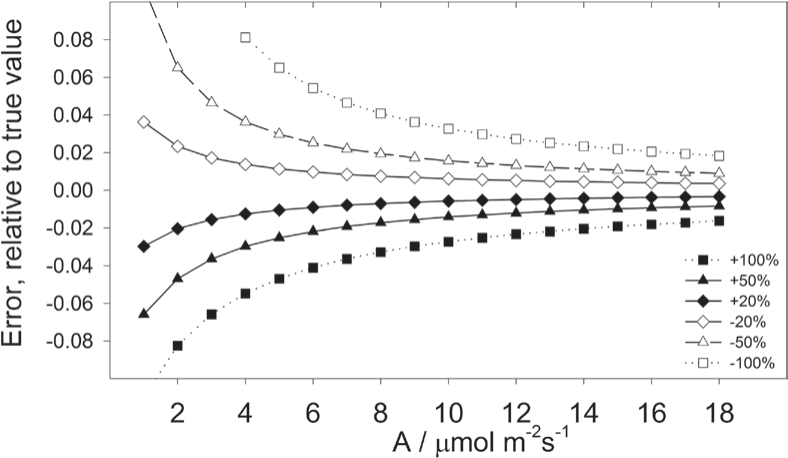
Sensitivity to errors in the determination of *R*_LIGHT_. True values were simulated by calculating equation 8 for *R*_LIGHT_=1 μmol m^–2^ s^–1^, *V*_O_/*V*_C_ = 0.2, and *Y(II)*=0.65 at variable assimilation (*A*) values. Test values of *V*_O_/*V*_C_ were then calculated by solving equation 8 at different values for *R*_LIGHT_: 2 μmol m^–2^ s^–1^ (+100%), 1.5 μmol m^–2^ s^–1^ (+50%), 1.2 μmol m^–2^ s^–1^ (+20%), 0.8 μmol m^–2^ s^–1^ (–20%), 0.5 μmol m^–2^ s^–1^ (–50%), 0 μmol m^–2^ s^–1^ (–100%, *GA*=*A*). The difference in *V*_O_/*V*_C_ between the test minus the true value was expressed as relative to the true value.

To account for the extent that *R*_LIGHT_ affected the measurement of *V*_O_/*V*_C_, a sensitivity analysis was used to determine how *R*_LIGHT_ influences *V*_O_/*V*_C_ (Fig. 3). To do so, equation 8 was calculated for a realistic dataset (*R*_LIGHT_=1 μmol m^–2^ s^–1^, *V*_O_/*V*_C_=0.2 and *Y(II)*=0.65) at variable assimilation values. Then, test values for *V*_O_/*V*_C_ were calculated after *R*_LIGHT_ was varied to 2 μmol m^–2^ s^–1^ (+100%), 1.5 μmol m^–2^ s^–1^ (+50%), 1.2 μmol m^–2^ s^–1^ (+20%), 0.8 μmol m^–2^ s^–1^ (–20%), 0.5 μmol m^–2^ s^–1^ (–50%), 0 μmol m^–2^ s^–1^ (–100%, *GA*=*A*). The deviation from the set *V*_O_/*V*_C_ value (0.2) represented the effect of errors in the evaluation of *R*_LIGHT_ on *V*_O_/*V*_C_. Figure 3 shows that *V*_O_/*V*_C_ was relatively insensitive to *R*_LIGHT_: for assimilation rates higher than 4 μmol m^–2^ s^–1^, *R*_LIGHT_ values which differed ± 50% resulted in an error lower than 4% in relative terms. *R*_LIGHT_ overestimation resulted in a lower error than *R*_LIGHT_ underestimation. For these reasons there is generally no need for a high quality estimate of *R*_LIGHT_.

### Accuracy of *Fm′* measurements

Equations 7 and 8 require the photochemical yield of PSII, *Y(II)*. This is determined according to the formula of Genty (Genty *et al.*, 1989; Maxwell and Johnson, 2000; Kramer *et al.*, 2004), whereby *Y(II)* is calculated as the difference between the light-saturated chlorophyll fluorescence signal (*Fm′*) minus the chlorophyll fluorescence signal measured during photosynthesis (*Fs*), expressed as relative to *Fm′*. Key to this technique is achieving full saturation of PSII in the determination of *Fm′* (Earl and Ennahli, 2004; Loriaux *et al.*, 2006; Harbinson, 2013; Loriaux *et al.*, 2013). Sub-saturating light pulses result in the underestimation of *Fm′*; however, the degree of underestimation depends not only on the saturating pulse spectra and intensity, but also on the species, the growth light intensity, and the light intensity used during the measurements (Earl and Ennahli, 2004).

**Fig. 4.**
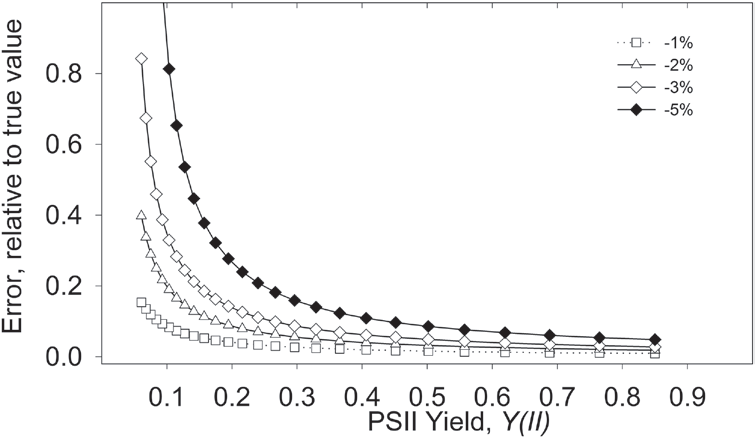
Sensitivity to errors in the determination of *Fm′*. True values were simulated by calculating equation 8 for *R*_LIGHT_=1 μmol m^–2^ s^–1^, *V*_O_/*V*_C_=0.2 and *A*=5 μmol m^–2^ s^–1^ at different *Y(II)* values. Test values of *V*_O_/*V*_C_ were then calculated by solving equation 8 introducing increasing *Fm′* underestimation: –1, –2, –3, and –5%. The difference in *V*_O_/*V*_C_ between the test minus the true value was expressed as relative to the true value.

Here, we show how a given *Fm′* underestimation influences the values for *V*_O_/*V*_C_ (Fig. 4). To do so, equation 8 was set to physiologically realistic conditions (*R*_LIGHT_=1 μmol m^–2^ s^–1^, *V*_O_/*V*_C_=0.2, and *A*=5 μmol m^–2^ s^–1^), at different *Y(II)* values. Underestimates of *Fm′* were then introduced by multiplying the realistic *Fm′* value by, successively, 0.99 (–1%), 0.98 (–2%), 0.97 (–3%), and 0.95 (–5%). The difference between the two values represented the effect of *Fm′* underestimation on *V*_O_/*V*_C_. Figure 4 shows that *V*_O_/*V*_C_ was sensitive to *Fm′* underestimation; for instance the relative error of *V*_O_/*V*_C_ was c. 20% when *Y(II)* was 0.15 and *Fm′* was underestimated by 3%. The error increased hyperbolically at decreasing *Y(II)*, and increased proportionally as the *Fm′* underestimation was increased.

### Light intensity and CO_2_ concentration used for experimentation

High light intensities (e.g. PPFD>1000 μmol m^–2^ s^–1^) result in a low PSII yield, which may potentially amplify the systematic error from any *Fm′* underestimation (see above). Similarly, small *Y(II)* could potentially lead to *V*_O_/*V*_C_ underestimation when the allocation to alternative sinks is significant (see description of equation 7). Further, high light conditions require longer timescales to reach stable photosynthetic conditions. On the other hand, depending on growth conditions, low light intensities (e.g. <100 μmol m^–2^ s^–1^) might lead to low assimilation rates, which could amplify the systematic errors in the estimation of *R*_LIGHT_ (see Fig. 3 and above). For these reasons, intermediate light intensities represent the best solution, whereby *Y(II)* and *A* are both high. For instance, values at the top end of the linear region of the *light* response curve would be ideal. These generally correspond to the growth light intensity.

CO_2_ concentration in the cuvette (*C*_a_) can be used to manipulate photorespiration. Figure 2 shows the measured and predicted *V*_O_/*V*_C_ of C_3_ and C_4_ plants under different CO_2_ concentrations. Because of the CCM, *V*_O_/*V*_C_ is low in maize, even at low *C*_i_, whereas in wheat *V*_O_/*V*_C_ increases hyperbolically at decreasing *C*_i_. This contrasting behaviour allows the resolution of the method to be manipulated by changing the CO_2_ concentration in the background gas. However, decreasing CO_2_ concentration is disadvantageous because: (i) low *C*_i_ results in quenching of PSII yield, which may potentially amplify the systematic error determined by *Fm′* underestimation (see above); at the same time (ii) low *Y(II)* would amplify the magnitude of *V*_O_/*V*_C_ underestimation owing to the partitioning of *Y(II)* to alternative sinks (see description of equation 7); (iii) under low *C*_a_, more time is required to reach stable photosynthetic conditions, which result in lower throughput; (iv) low *C*_a_ increases the driving force for diffusion from outside of the cuvette, which may constitute a potential source of error, especially when assimilation is low (Boesgaard *et al.*, 2013). For these reasons the optimal *C*_a_ will depend on the purpose of the analysis, and on the desired resolution and speed.

## Discussion

This method is based upon the difference in net assimilation (*A*) and photosystem II yield (*Y(II)*) observed when the gas supplied to an actively photosynthesizing leaf is switched from ambient O_2_ to low O_2_. The goal was to develop a relatively quick, readily available method, which could be used to screen large numbers of transformants, C_3_–C_4_, C_2_, or photorespiratory refixation variants (Busch et al 2013; Oakley *et al.*, 2014) in a given population of plants. The data show that the method readily distinguishes between *V*_O_/*V*_C_ for typical C_3_ and C_4_ plants (Table 2), and, given the low coefficients of variation, should detect more subtle variations in C_4_ repression or activation within a screen. It would then be possible to subject plants identified in this way to a more detailed, conventional gas exchange or stable isotope screen, to identify contributory morphological, metabolic or genetic factors. In the subsequent discussion, we explore the theoretical and practical limitations underpinning the accuracy of the method, and improvements that could be instituted to enhance the outputs, if high sample throughput was not a primary limitation.

Other methods have been proposed to determine the contribution of photorespiration *in vivo* through gas exchange measurements. The method proposed by Ripley *et al.* (2007) uses only the increase in assimilation under non-photorespiratory conditions, and therefore ignores the effect on *Y(II)*. In our work we observed that *Y(II)* is generally influenced by changes in O_2_ concentration (Figure 1), even in C_4_ plants (see Fig. 2 in Bellasio and Griffiths, 2014b); therefore it is important to take into account the feedback from assimilation on photosystem II yield. Long and Bernacchi (Long and Bernacchi, 2003) proposed a comprehensive method to determine the partitioning of total electron transport rate between photorespiratory and assimilatory demand. Their protocol requires an initial light or A/C_i_ response so as to fit a linear relationship between quantum yield for CO_2_ fixation *Y(CO*_2_) and quantum yield of photosystem II, *Y(II)*.

In comparison, the simple method that we have proposed requires no previous parameterization, no curve fitting, and no knowledge of the underpinning physiology or biochemical constants. It is also independent of leaf area, as when deriving *V*_O_/*V*_C_ from equation 8, both the numerator and the denominator are proportional to leaf area, a huge advantage for small or dissected leaves. The likelihood of triose phosphate limitation (Sharkey, 1988) is minimized under the relatively low light intensities and low *C*_i_, which are optimal for this protocol. The determination of *V*_O_/*V*_C_ could take as little as c. 6min, although the complete routine was longer (c. 40min) as leaves were allowed to acclimate before measurement of both assimilation and dark respiration. Therefore, the run time can be minimized by measuring assimilation under growth conditions (e.g. at growth light intensity and CO_2_ concentration), and either measuring respiration after all plants have been collectively dark–adapted, or estimating it separately (see below).

### Other factors affecting accuracy of *V*_O_/*V*_C_ determination

As shown in Fig. 3, the estimation of *R*_LIGHT_ is important when calculating gross assimilation using eqn. 8 (*GA*=*A*+*R*_LIGHT_) at low assimilation rates. *R*_LIGHT_ can be determined with several methods; for instance, by linear regression of assimilation (*A*) versus irradiance (under very low irradiance e.g. <150 μmol photons m^–2^ s^–1^), by linear regression of *A* versus irradiance multiplied by *Y(II)* [under moderate irradiance, e.g. <400 μmol photons m^–2^ s^–1^ (Yin *et al.*, 2011a)], by non-linear regression [throughout the light response curve (Prioul and Chartier, 1977; Dougherty *et al.*, 1994)] or assumed to equal dark respiration [e.g. (Kromdijk *et al.*, 2010; Ubierna *et al.*, 2013)]. These methods do not necessarily yield the same *R*_LIGHT_ values, and so, the degree of similarity between different *R*_LIGHT_ estimates depends on the species and growth conditions. For instance, in Cocklebur (*Xanthium strumarium* L., Asteraceae), *R*_LIGHT_ was significantly different from dark respiration (Tcherkez *et al.*, 2008), whereas in maize *R*_LIGHT_ is generally non-significantly different from dark respiration (C. Bellasio, unpublished data). The most suitable method to estimate *R*_LIGHT_ should therefore be evaluated on a case-by-case basis (see Bellasio and Griffiths, 2014a), and for a uniform population (e.g. one species or set of transformants in a growth chamber), *R*_LIGHT_ could be estimated on a subset of individuals, with one of the methods described above. If dark respiration is used as a proxy, the quality of the estimate can be increased using large chambers and low flow rates. In a diverse population, *R*_LIGHT_ could be estimated by measuring dark respiration on each individual plant after the measurements in the light.

As shown in Fig. 3, errors in the determination of *Fm′* suggest that techniques such as the multiphase flash (Loriaux *et al.*, 2013), or initial checks to ensure that the saturating pulse is saturating (see Bellasio and Griffiths, 2014b) are normally appropriate for this method. However, the use of our method is possible without a multiphase flash. Firstly, the underestimation of *Fm′* introduces a systematic error, i.e. comparable plants will normally show similar *V*_O_/*V*_C_ (see Bellasio *et al.*, 2014), unless the extent of C_4_ or C_2_ activity has changed under these conditions. Thus, the precision and the resolution of the method, when comparing different phenotypes against a common genetic background, are not affected by a consistent underestimation of *Fm′*. Secondly, to improve accuracy, i.e. the capacity of the method to estimate the true *V*_O_/*V*_C_, other approaches could: (i) increase the saturating pulse intensity; (ii) reduce the distance between light source or fibre-optic probe and leaf (in some systems); (iii) decrease actinic light intensity (as shown in this study) to maximise *Y(II)*; and (iv) CO_2_ concentration can be increased, in order to maximise *Y(II).*

### IRGA recalibration, matching *Y(II)*, *C*_i_, and consideration of mesophyll conductance

As mentioned in the results, a slight effect on stomatal conductance and *C*_i_ (under low O_2_) could have been caused by not recalibrating the IRGA upon switching background gas (Bunce, 2002). Although that recalibration could have increased *C*_i_ and *g*_S_ accuracy (under low O_2_), this procedure is liable to introduce operator error and extend the time taken for measurements; further, there are theoretical reasons why we need not account for these processes while carrying out such a simple comparative screen. Firstly, the data used to calculate equation 8 are measured by the CO_2_ channel of the IRGA and the fluorometer, which are both unaffected by the background gas (Bunce, 2002). Secondly, the effect of *C*_i_ on *A* (under low O_2_) is, for the greatest part, accounted by the feedback on Y(II).

Although *C*_i_ decreases under low O_2_, there is a strong feedback between assimilation and *Y(II)*, and therefore *Y(II)* decreases proportionally. In fact, the relationship between gross assimilation (or, better, between *Y(CO*_2_), which is *GA* divided by PPFD) and *Y(II)* is strictly linear (Edwards and Baker, 1993; Valentini *et al.*, 1995; Martins *et al.*, 2013). In C_4_ plants, this linear relationship has generally a zero intercept, (Edwards and Baker, 1993); therefore, for C_4_ plants, there is no need for curve fitting and the relationship can be correctly estimated with a single point. In C_3_ systems this relationship is still linear but the intercept is, although generally small, not zero. The intercept, which is the magnitude of engagemant of alternative sinks, can be estimated by linear curve fitting, although several data points are required (Valentini *et al.*, 1995; Martins *et al.*, 2013). Using the complete fitting of the *Y(CO*_2_)/*Y(II)* relationship, however, did not improve the estimate of *V*_O_/*V*_C_ (data not shown): the complete curve fitting correctly estimates the intercept, but the datapoints are taken under conditions which differ from those under which *V*_O_/*V*_C_ is measured.

Another way to improve the estimate of *V*_O_/*V*_C_ would be to adjust *C*_a_ under low O_2_ so as to match *Y(II)* measured under ambient O_2_ with *Y(II)* measured under low O_2_. Alternatively, *C*_a_ could be manipulated to deliver *C*_i_ under low O_2_, which matches that under ambient. The advantages would be that the measured data would then probably fit the predicted C_3_ and C_4_ models more precisely when *C*_i_ is limiting (see Fig. 2, Tables 3 and 4). However, these operations do not improve the capacity to screen between C_3_ and C_4_ photosynthesis and the additional manipulations increase time and likelihood of errors. We also note that such improvements would allow this method to be used to calculate the CO_2_ concentration at the site of carboxylation (*C*_C_) in C_3_ plants through equation 11 (Table 3), as well as mesophyll conductance via equation 10, using *C*_C_, and the values for assimilation and *C*_i_ measured under ambient conditions.

## Conclusion

In this paper a simple method, and associated theory, have been presented, which allow the determination of both the oxygenation (*V*_O_) and carboxylation (*V*_C_) rate of RuBisCO and the rate of photorespiratory CO_2_ evolution (*F*) based on gas exchange and variable chlorophyll fluorescence under ambient and low O_2_. This may be of particular interest for high throughput screening to identify C_4_ mutants lacking a fully functional CCM, C_2_ variants, or populations of C_3_–C_4_ hybrids (Oakley *et al.*, 2014).
